# Periodontal Application of Manuka Honey: Antimicrobial and Demineralising Effects In Vitro

**DOI:** 10.1155/2017/9874535

**Published:** 2017-03-14

**Authors:** Syarida H. Safii, Geoffrey R. Tompkins, Warwick J. Duncan

**Affiliations:** ^1^Sir John Walsh Research Institute, Faculty of Dentistry, University of Otago, Dunedin 9016, New Zealand; ^2^Department of Restorative Dentistry, Faculty of Dentistry, University of Malaya, 50603 Kuala Lumpur, Malaysia

## Abstract

*Background*. Topical application of manuka honey is effective in the treatment of burns and soft-tissue infections. The aim of this study was to assess the antibacterial activity of manuka honey against plaque-associated bacteria in vitro in order to evaluate the potential application as an adjunct to periodontal treatment.* Materials and Methods*. The minimum bacteriostatic and bactericidal concentrations (MIC and MBC) of manuka honey were compared to those of white clover honey against a variety of plaque-associated bacteria, at the natural and neutral pH. Dissolved calcium was measured following incubation of honeys with hydroxyapatite (HA) beads to assess their potential to demineralise oral hard tissues.* Results*. Both honeys inhibited most tested oral bacteria at similar MIC/MBC, but* Streptococcus mutans* was comparatively resistant. The honeys at pH neutral had little effect on antimicrobial activity. Incubation of HA beads in honey solutions resulted in pH-dependent calcium dissolution, and inoculation with* S. mutans* promoted further demineralisation by both types of honey.* Conclusion*. Manuka honey is antimicrobial towards representative oral bacteria. However, the relative resistance of* S. mutans* in association with the high concentrations of fermentable carbohydrates in honey and the direct demineralising effect at natural pH mitigate against the application of honey as an adjunct in the treatment of periodontal disease.

## 1. Introduction

Honey has been used since ancient times in many cultures to treat infections and other medical conditions [1, 2]. Since the 1990s, research on honeys has regained momentum, with focus towards antibacterial properties particularly against bacteria associated with antibiotic-resistance and infected wounds [3–5]. This has led to approval for use of manuka honey in the treatment of infected wounds, burns, and ulcers [6–9]. Furthermore, with increasing bacterial resistance to antimicrobials used in periodontal therapy [10, 11], there is a requirement to explore alternatives to conventional antibiotics.

The antibacterial factors in honey include the hyperosmolarity effect (>80% sugar content), acidic pH, hydrogen peroxide, methylglyoxal, bee defensin-1, various proteinaceous compounds, flavonoids, and phenolic compounds [4, 12, 13], but the principal antimicrobial activity of most honeys is due to hydrogen peroxide [14]. Medical-grade manuka honey, derived from the shrub* Leptospermum scoparium* (native to New Zealand and Australia), contains unusually high concentrations of methylglyoxal and only trace amounts of hydrogen peroxide [12, 13, 15].

Nonperoxide antibacterial activity (NPA) (commercially registered as Unique Manuka Factor [UMF®]) indicates the antibacterial efficacy against* Staphylococcus aureus,* expressed as the equivalent phenol concentration [16]. The antimicrobial activity of honeys varies depending on botanical, geographical, and seasonal conditions. In New Zealand, manuka honey with elevated antimicrobial activity is harvested from select regions in the North Island, particularly East Cape, Waikato, and Bay of Plenty [17].

Manuka honey has antimicrobial activity in vitro towards a variety of bacteria including dental plaque-associated bacteria, both as planktonic and biofilm organisms [18–20].* Porphyromonas gingivalis,* which is associated with various periodontal diseases [21], and* Aggregatibacter actinomycetemcomitans, *associated with aggressive periodontitis [22], are sensitive to manuka honey when grown as planktonic bacteria [23, 24] but* P. gingivalis* is considerably more resistant when cultivated as a biofilm [25]. English et al. [26] reported reduced plaque accumulation and gingival bleeding among human participants with gingivitis after chewing manuka honey strips for ten minutes daily for 21 days, suggesting beneficial application of manuka honey as an oral health aid.

The aim of this study was to assess the antibacterial activity of medical-grade manuka honey in vitro against dental plaque-associated bacteria. Manuka honey has a pH < 4 and is principally composed of fermentable carbohydrate which may mitigate against direct application adjacent to mineralised oral tissues. Thus, to explore the potential of manuka honey as a locally delivered subgingival clinical adjunct in the treatment of chronic periodontitis, we also evaluated the demineralising potential of honey.

## 2. Materials and Methods

### 2.1. Bacterial Strains and Culture Conditions

*Staphylococcus aureus* Oxford,* Escherichia coli* DH5*α*,* Streptococcus mutans* UA159,* Streptococcus mutans* ATCC 10449,* Streptococcus sobrinus* OMZ 176,* Streptococcus sanguinis* ATCC 10556,* Streptococcus gordonii* ATCC 10558,* Fusobacterium nucleatum* ATCC: 10953, 25586, 33568, 49256;* Porphyromonas gingivalis* ATCC 33277, and* Prevotella intermedia* ATCC 25611 were from the University of Otago culture collection.* Staph. aureus* and* E. coli* were included as reference gram-positive and gram-negative bacteria, respectively [27, 28].

*Staph. aureus* and* E. coli* were cultured in tryptic soy broth (TSB) (BactoTM, Becton Dickinson Co., Sparks, MD, USA) and on TSB agar, under aerobic conditions. Streptococci were cultured on Columbia sheep-blood agar (CBA) (Fort Richard, Mt. Wellington, New Zealand) under anaerobic conditions (10% H_2_, 5% CO_2_, and 85% N_2_) in a MACS work station (MG500, Don Whitley Scientific Ltd., Shipley, United Kingdom) and were grown in brain heart infusion (BHI) (Difco Laboratories, Detroit, MI, USA).* F. nucleatum*,* P. gingivalis*, and* P. intermedia* were cultured and maintained under anaerobic conditions on CBA and in prereduced BHI supplemented with hemin chloride (5 *μ*g/ml; Sigma Chemical Co., St. Louis, MO, USA) and menadione (1 *μ*g/ml; Sigma). All incubations were at 37°C.

### 2.2. Honey Preparations

Medical-grade manuka honey (NPA20+ and NPA25+, Comvita New Zealand Ltd., Bay of Plenty, New Zealand) and nonmedical grade white clover honey (Hollands NZ Honey Ltd., Timaru, New Zealand) were purchased from local retailers. Each honey was dissolved in culture medium to 50% (w/v), filter-sterilised (0.22 *μ*m; Millex®GP, Merck Millipore Ltd., Carrigtwohill, Ireland), and further diluted in sterile medium. Honey/culture medium preparations were adjusted to pH 7.1 (±0.05) by adding calcium hydroxide (Sigma Aldrich, St. Louis, MO, USA) using a microprocessor pH meter (pH211 with HI 1230B electrode, Hanna Instruments, Woonsocket, RI, USA). The pH-adjusted honey preparations were filter-sterilised and diluted in culture media.

### 2.3. Bacteriostatic and Bactericidal Assays

Minimum inhibitory concentration (MIC) and minimum bactericidal concentration (MBC) assays were performed according to CLSI reference methods for bacteria [29]. Assays were prepared in 96-well flat-bottomed microtitre trays, each well containing 200 *μ*L of honey preparation inoculated with 10 *μ*L of bacterial culture adjusted to a McFarland standard of 0.5. The MICs were determined by optical density (*A*_600_) measurement (Biotek Instruments Synergy 2, Vermont, NE, USA) and MBCs by spot-plating 10 *μ*L onto appropriate agar followed by incubation (as above). MBC was determined as the lowest concentration resulting in complete killing of the test bacterium. The MBC assay was adapted to determine the rapidity of killing by removing and plating samples at specific times.

### 2.4. Demineralisation Assay

The honeys were dissolved in distilled water at 50% (w/v), and 1 mL was added to 10 mg of hydroxyapatite (HA) beads (particle size of 80 *μ*m; Bio-Rad Laboratories, Hercules, CA, USA). Hydrochloric acid (0.1 M) and distilled water were used as positive and negative demineralisation controls, respectively. After incubation for 24 h, supernatants were recovered and solubilised calcium measured with a colorimetric detection assay kit (Abcam Australia Pty Ltd., Melbourne, Australia) according to the manufacturer's directions. Honey preparations (without HA beads) were assayed to determine intrinsic calcium concentrations which were subtracted from the demineralisation assays.

The demineralisation assay was modified to assess the additional influence of* S. mutans*. Honey dilutions were prepared in TSB without dextrose (TSB w/o D; Difco) and 1 mL was added to each tube containing sterilised HA beads (10 mg). An aliquot (50 *μ*L) of* S. mutans* (grown for 18 h in TSB w/o D) was used to inoculate the honey/HA preparations which were incubated for 24 hours. Supernatant samples were analysed for solubilised calcium.

## 3. Results

### 3.1. Bacteriostatic and Bactericidal Activities of Honeys

**Figure 1:**
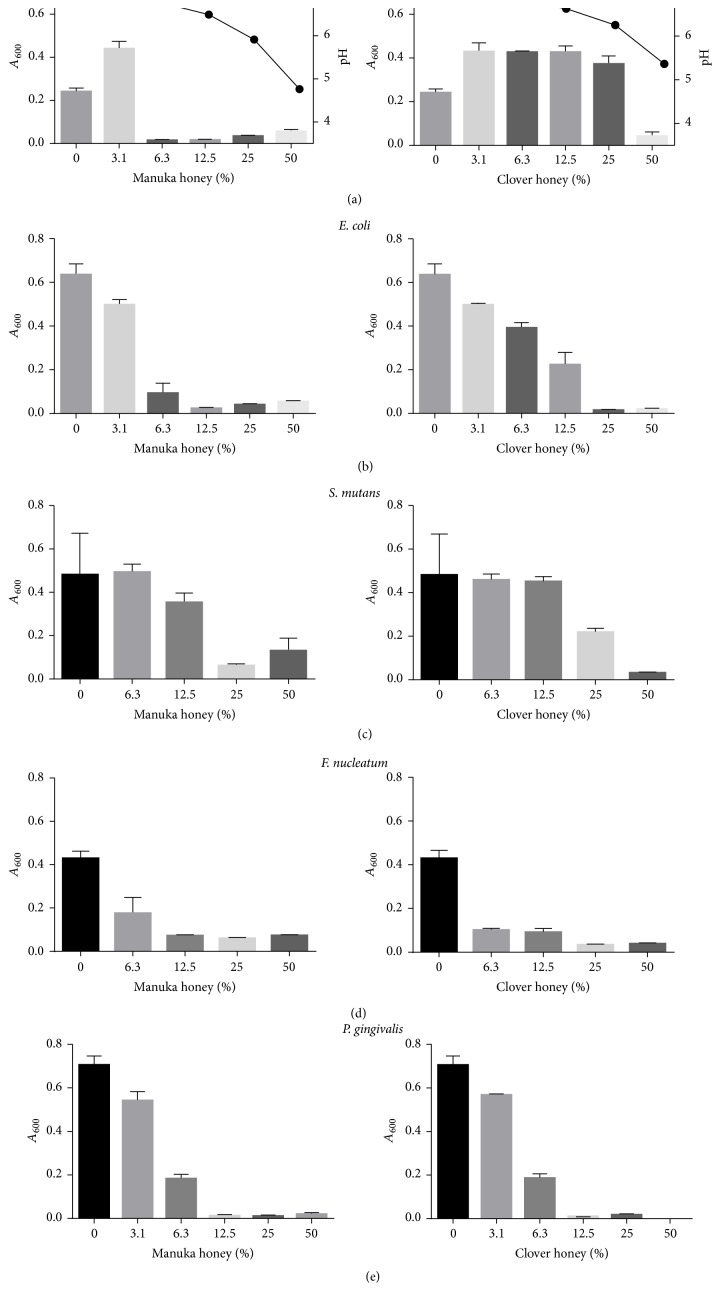
Minimum inhibitory concentrations (% w/v) of manuka and white clover honeys against various bacteria after 18 h incubation. pH of honey dilutions is recorded (a). Bacterial growth was determined as culture density (*A*_600_) in a microtitre-plate reader.* F. nucleatum*: ATCC 25586.

Neither manuka nor clover honey exhibited antibacterial effects within four-hour exposure to bacteria. With the exception of* S. mutans*, all tested bacteria were inhibited by both types of honey after 18 h incubation (Figure 1). Manuka honey displayed slightly greater inhibitory efficacy, with MICs ranging between 6.3% and 25%, whereas the MICs of clover honey ranged from 6.3% to 50% (Figure 1). Manuka honey was slightly more acidic than clover honey (Figure 1).

**Figure 2:**
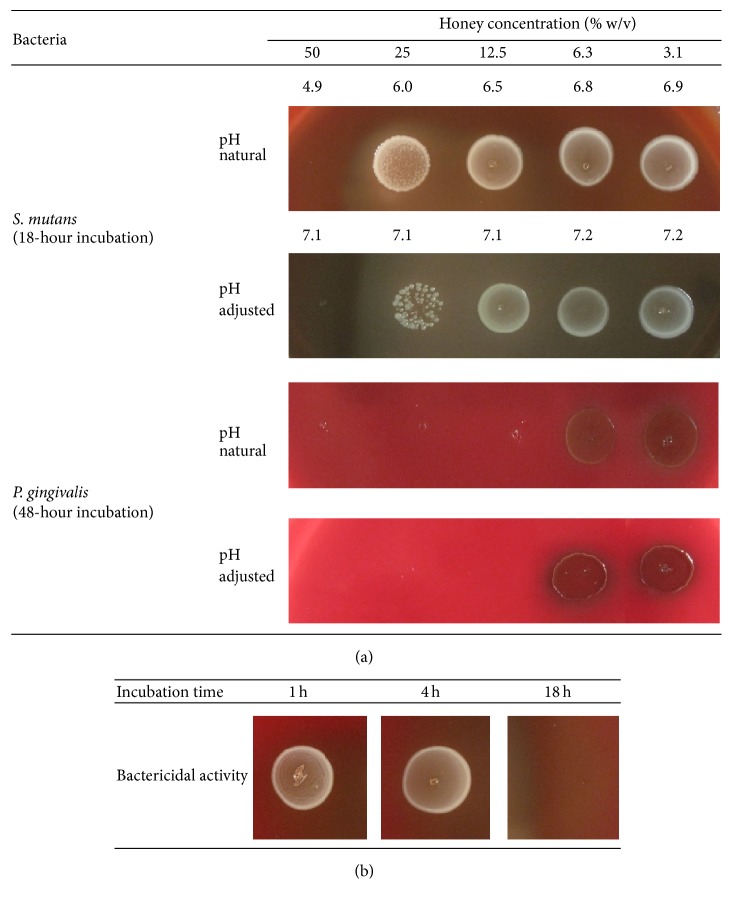
Bactericidal effect of manuka honey (NPA 25+) against (a)* S. mutans* and* P. gingivalis* and (b) effective incubation time of manuka honey [50% (w/v)] against* S. mutans*. NPA = nonperoxide antibacterial activity.

The MBCs of manuka honey showed a range of between 12.5% and 50% (w/v) and clover honey from 6.3% to 50% (Table 1; Figure 2).* S. mutans* was the most resistant of the tested species against both types of honey (Table 1) but was killed by manuka honey (NPA25+) at 50% (Figure 2). The bactericidal activity of both types of honey required 18 h exposure (Table 1; Figure 2). Following adjustment of the pH to neutrality, the bactericidal activity of manuka honey was largely maintained (Table 1; Figure 2(a)) whereas pH-adjusted clover honey exhibited varying bactericidal activity (Table 1).

### 3.2. Demineralisation of HA Beads Incubated in Honey

**Figure 3:**
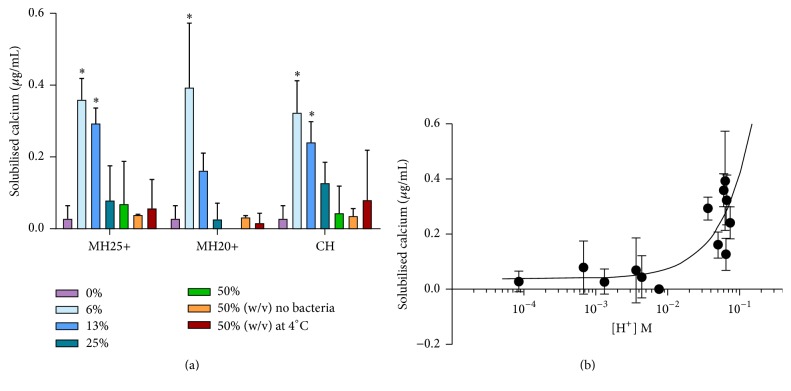
(a) Solubilisation of calcium from HA beads following incubation of* S. mutans* with manuka and clover honeys at 37°C and 4°C. ^*∗*^Significantly different from water control (*p* < 0.05; one-way ANOVA with Dunnett's post hoc test). (b) Relationship between solubilised calcium and final proton concentration. Correlation between calcium solubilisation and [H^+^] (*r*_*s*_ = 0.770, *p* < 0.01) Spearman's rho test. MH25+: manuka honey NPA25+; MH20+: manuka honey NPA 20+; CH: clover honey.

Incubation of HA beads in dilute honey resulted in significant solubilisation of calcium as compared to incubation in water. The two types of honey did not differ significantly in this respect (Figure 3(a)). Demineralisation also occurred when honey/HA preparations were maintained at 4°C (Figure 3(a)), suggesting that the effect was due to low pH rather than biological activity.

When the assay was modified by the addition of* S. mutans*, both types of honey promoted significant demineralisation at lower concentrations (Figure 3(a)) and calcium solubilisation was positively correlated to [H^+^] (*p* < 0.01) (Figure 3(b)). Decalcification decreased as the honey concentrations increased, due to the antibacterial effects of the honey (Figure 3(a)).

## 4. Discussion

A few clinical studies have reported reduction in plaque accumulation and gingival bleeding [26, 30] and decreased proportion of mutans streptococci [31] with application of honey intraorally, supporting the use of honey as an oral antimicrobial agent. Thus, the present preclinical study assessed the potential for subgingival application of manuka honey in the treatment of periodontal diseases.

Both manuka and clover honeys were active against a variety of organisms including plaque-associated bacteria; manuka honey was more active in this respect. In agreement with other studies [24, 28, 32],* P. gingivalis*,* P. intermedia*, and* F. nucleatum*, gram-negative bacteria associated with gingivitis and periodontal diseases, were more sensitive than the gram-positive species associated with gingival health (streptococci). Subgingival application of manuka honey could therefore conceivably promote health-associated species at the expense of those associated with periodontal diseases. However,* S. mutans* is generally considered cariogenic [33, 34] and was the most resistant of the tested organisms to both types of honey. As honey is principally composed of fermentable carbohydrates including fructose, glucose, and sucrose [35], localised application of manuka honey could conceivably select and encourage cariogenic species. Honey is considered cariogenic when consumed as a foodstuff [36].

As* S. mutans* is notably acid-tolerant [37, 38], the question arises as to whether the low pH of honey contributes to the antimicrobial effects against other species but adjusting the pH to neutrality had limited effect on the antimicrobial activity. The natural pH of honey ranges from 3.1 to 4.5 [12, 39], well below the accepted critical pH of 5.5 for enamel and more importantly cementum demineralisation [40, 41] further mitigating against subgingival application of honey to root surfaces as an adjunct in the treatment of periodontitis. On the other hand, the evidence for enamel demineralisation following prolonged in vitro exposure to honey is contradictory [42–44]. Dilute honey should pose less of a threat to mineralised tissues but would also have less antimicrobial activity. Our in vitro findings demonstrate that diluting the honey not only reduces the antimicrobial activity but also promotes bacterial carbohydrate metabolism and consequent acid production by* S. mutans*, resulting in further demineralisation.

Pharmacokinetic studies of subgingivally delivered antimicrobial agents report exponential concentration reduction following application [45, 46], implying that the bacteriostatic action of honey will begin to diminish immediately following delivery to a periodontal pocket. Application of undiluted honey may have an effective initial antimicrobial effect but the low pH will facilitate demineralisation of adjacent cementum and, as the honey concentration falls, acid production by the surviving bacteria (i.e., fermentative streptococci) will promote further demineralisation.

Despite evidence that manuka honey provides effective antimicrobial effects when applied to soft tissues and encouraging clinical outcomes for intraoral use, our findings suggest that it could be damaging to calcified tissues. Nevertheless, whether a single application (rather than a sustained release) would deliver a beneficial antimicrobial effect without threatening root integrity remains to be determined. However, other beneficial oral applications of manuka honey, which do not threaten the mineralised tissues, might be considered, for example, to dental implants in the treatment of peri-implantitis.

## 5. Conclusion

Medical-grade manuka honey (i.e., NPA > 20) is antimicrobial towards representative oral bacteria generally, and the gram-negative anaerobes associated with gingivitis are particularly sensitive. However, the relative resistance of cariogenic* S. mutans* in association with the high concentrations of fermentable carbohydrates in honey and the direct demineralisation of oral hard tissues caused by the low pH of honey mitigate against application as a subgingival sustained-release adjunct in the treatment of periodontal disease.

## Figures and Tables

**Table 1:** Minimum bactericidal concentrations (MBC) of manuka (NPA 20+) and white clover honeys against a variety of bacteria.

Bacteria	MBC (% w/v)			
	Manuka honey NPA20+		White clover honey	
	pH 4.9^*∗*^	pH 7.1	pH 5.2^*∗*^	pH 7.1
*Staph. aureus*	12.5	12.5	50	>50
*E. coli*	12.5	25	25	50
*S. mutans UA159*	>50	>50	>50	>50
*S. mutans 10449*	25	25	25	50
*S. sobrinus *	12.5	12.5	25	25
*S. sanguinis*	25	25	50	50
*S. gordonii*	25	25	25	>50
*F. nucleatum* ATCC 25586	12.5	12.5	25	25
*F. nucleatum* ATCC 33568	25	25	25	25
*P. gingivalis*	12.5	12.5	12.5	25
*P. intermedia*	12.5	12.5	25	12.5

*P. gingivalis* and *P. intermedia* were incubated for 48 h while all other bacteria were incubated for 18 h. The highest honey concentration tested was 50% (w/v).

NPA: nonperoxide antibacterial activity.

^*∗*^Natural pH at 50% (w/v) in culture media.
